# Oxadiazole-2-oxides may have other functional targets, in addition to SjTGR, through which they cause mortality in *Schistosoma japonicum*

**DOI:** 10.1186/s13071-016-1301-3

**Published:** 2016-01-20

**Authors:** Li-Jun Song, Huan Luo, Wen-Hua Fan, Gu-Ping Wang, Xu-Ren Yin, Shuang Shen, Jie Wang, Yi Jin, Wei Zhang, Hong Gao, Qian Liu, Wen-Long Wang, Bainian Feng, Chuan-Xin Yu

**Affiliations:** Key Laboratory on Technology for Disease Prevention and Control, Ministry of Health, Jiangsu Provincial Key laboratory of Parasite Molecular Biology, Jiangsu Institute of Parasitic Diseases, Wuxi, Jiangsu 214064 China; Public Health Research Center, Jiangnan University, Wuxi, 214122 China; School of Pharmaceutical Science, Jiangnan University, Wuxi, 214122 China; Medical College, Jiangnan University, Wuxi, 214122 China

**Keywords:** *Schistosoma japonicum*, Oxadiazole-2-oxide, Antischistosomal activity, Structure-activity relationship (SAR)

## Abstract

**Background:**

Schistosomiasis is one of the world’s major public health problems. Besides praziquantel (PZQ), there is currently no other effective treatment against schistosomiasis. The development of new antischistosomal agents to curb the emergence of PZQ resistance should be a high priority. Oxadiazole-2-oxides have been identified as potential antischistosomal reagents, with thioredoxin glutathione reductase (TGR) being one of their molecular targets.

**Methods:**

To develop novel treatment reagents against *Schistosoma japonicum*, 30 novel oxadiazole-2-oxides were synthesised and their antischistosomal activities on juvenile and adult *S. japonicum* were evaluated *in vitro* and *in vivo*. Their inhibitory activities against *S. japonicum* thioredoxin glutathione reductase (SjTGR) were also analysed.

**Results:**

Most of the oxadiazole-2-oxides showed good juvenile and adult *S. japonica* killing activities *in vitro*. However, the antischistosomal effects of these compounds were not positively correlated with either their inhibition of SjTGR, or with nitric oxide (NO) release. Compounds **4a**, **4b**, **7c**, **13**, **16** and **20** resulted in 87.7 %, 83.1 %, 87.1 %, 84.6 %, 90.8 % and 69.5 %, respectively, mortality in the adult worms, when used to treat infected mice at schistosomula stage. These mortality rates were similar to or higher than that of artemisinin. Furthermore, compounds **4a** and **16** resulted in 66.7 % and 69.4 % reductions in the worm burdens, respectively, when infected mice were treated at the adult worm stage. These treatment effects were similar to PZQ. No differences in activity of the oxadiazole-2-oxides against female and male adult worms were observed. The toxicity of the oxadiazole-2-oxides on mammalian cells appeared to be similar to, or less than, that of PZQ.

**Conclusions:**

The antischistosomal activity of the oxadiazole-2-oxides does not depend on NO production or the inhibition of SjTGR activity. There may be other functional targets of the oxadiazole-2-oxides in *S. japonicum*. Several of the novel oxadiazole-2-oxides synthesised in this study could be used to develop novel antischistosomal drugs and explore potential molecular targets.

**Electronic supplementary material:**

The online version of this article (doi:10.1186/s13071-016-1301-3) contains supplementary material, which is available to authorized users.

## Background

Schistosomiasis is one of the most prevalent parasitic diseases. It has been estimated that 779 million people are at risk of infection worldwide [1]. The infection is caused by trematodes of the genus *Schistosoma*, among which *Schistosoma mansoni*, *S. haematobium*, *S. japonicum, S. mekongi* and *S. intercalatum* represent the most important pathogenic species for human beings. Depending on the species, the worms (schistosomes) persist in the liver and hepatic portal system or the urinary tract system of humans. Mature schistosomes lay eggs within their host, which often get trapped in the host’s tissues, resulting in inflammatory and obstructive diseases of the affected organs [2]. In China, the disease caused by *S. japonica* is a major public health concern, with more than 280 thousand people infected [3]. To cure subtle morbidity and prevent the development of severe chronic stage hepatosplenomegaly, praziquantel (PZQ) is the only choice of chemotherapy. The drug has been widely used for more than three decades and is therefore susceptible to the emergence of praziquantel-resistant schistosomes [4]. In addition, the mechanism by which PZQ works is still undefined [5].

A number of recent studies have attempted to modify PZQ [6–12], explore new chemical entities from natural products [13] and identify additional drug targets for the chemotherapy of schistosomiasis [14–18]. Oxadiazole-2-oxides were identified as new drug leads against *S. mansoni*, while thioredoxin glutathione reductase (TGR) was found to be a potential molecular target of the oxadiazole-2-oxides [15, 16]. Previously, we have shown that *S. japonicum* TGR (SjTGR) plays an essential role in maintaining the redox balance in *S. japonicum* and confirmed SjTGR as a potential target for the development of new drugs against schistosomiasis [18].

To our knowledge, few data have been reported for oxadiazole-2-oxides against *S. japonicum* [16]. We designed and synthesised novel oxadiazole-2-oxides to target SjTGR. The results show that some novel oxadiazole-2-oxides had a good killing activity against *S. japonicum*, but did not inhibit the activity of SjTGR, implying that oxadiazole-2-oxides may have other functional targets in *S. japonicum*.

## Methods

### Reagents

5,5’-Dithiobis-(2-nitrobenzoic acid) (DTNB) and β-nicotinamide adenine dinucleotide 2’-phosphate reduced tetrasodium salt (NADPH) were purchased from Roche (Basel, Switzerland). RPMI 1640 and fetal bovine serum were from Gibco (Invitrogen Corporation, Carlsbad, USA). 3-(4,5-dimethyl-2-thiazolyl)-2,5-diphenyl-2-H-tetrazolium bromide (MTT), dimethyl sulfoxide (DMSO) and PZQ were sourced from Sigma-Aldrich Inc. (Saint Louis, USA). The Nitric Oxide Assay Kit and 2-(4-carboxyphenyl)-4,4,5,5-tetramethylimidazoline-1-oxyl-3-oxide (carboxy-PTIO) came from Beyotime Biotechnology Corporation (Shanghai, China). Ethylenediaminetetraacetic acid (EDTA) and 2-[4-(2-hydroxyethyl)-1-piperazinyl]ethanesulfonic acid (HEPES) were purchased from Sangon Biotech Co. Ltd. (Shanghai, China), and artemisinin (AS) was purchased from Zelang Medical Technology Co. Ltd. (Nanjing, China).

### Parasites and experimental animals

Infected *Oncomelania hupensis*, provided by the Department of Snail Biology, Jiangsu Institute of Parasitic Diseases, were incubated at 25–28 °C, under illumination, for 2 h to hatch *S. japonicum* cercariae. The cercariae were collected from the water surface with clean cover glass and placed into a centrifuge tube using pre cooled RPMI 1640. Centrifugation was performed for 1 min at 1500 rpm, and then the steps above were repeated. After centrifugation, the supernatant solution was removed. The cercariae at the bottom were washed twice and resuspended with RPMI 1640. Approximately 20–30 cercariae (1 mL of the resuspension medium) were added to one well in a 24-well culture plate. They were incubated overnight at 37 °C with 5 % CO_2_. The survival rates and number of tails shed were observed under an inverted microscope. Cercariae that had shed their tails were used as juvenile *S. japonicum* [19, 20].

ICR mice (20–22 g), were obtained from the Shanghai Sub-Center of Experimental Animals, Chinese Academy of Sciences. They were maintained at the Department of Experimental Animals, Jiangsu Institute of Parasitic Diseases. To infect each mouse, its abdominal skin was exposed to 50 *S. japonica* cercariae. All of the mice were executed to collect *S. japonica* adult worms, through portal vein perfusion, 35 days post-infection [21]. The adult *S. japonica* collected were washed with RPMI 1640 medium and then kept in RPMI 1640 medium (pH 7.5) with HEPES 20 mM, penicillin (100 IU/mL), streptomycin (100 mg/mL) and 10 % fetal bovine serum [22]. Then, two pairs of adult worms were transferred into each well of a 24-well culture plate, with 2 mL of the same medium. The worms were cultured at 37 °C in a humid atmosphere with 5 % CO_2_.

### Synthesis of oxadiazole-2-oxide derivatives

**Scheme 1:**
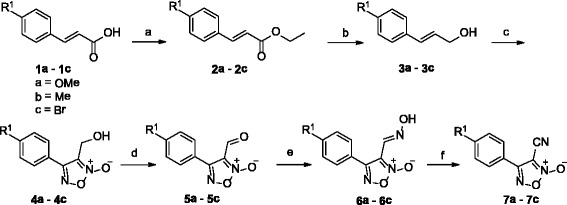
Synthesis of compounds **4a**–**4c** and **7a–7c**. *Reagents and conditions*: (a) EtOH, H_2_SO_4_, reflux, overnight; (b) DIBAL-H, DCM, N_2_, −70 °C to rt, 12 h, 90–95 %; (c) NaNO_2_, HOAc, H_2_O, 0 °C to rt, 25–32 %; (d) MnO_2_, DCM, water–ice to rt, 4 h, 92 %; (e) NH_4_OH-HCl, pyridine, EtOH, 90 °C, 3 h, 49 %; (f) SOCl_2_, DMF, N_2_, 30 °C, 12 h, 70–75 %

**Scheme 2:**
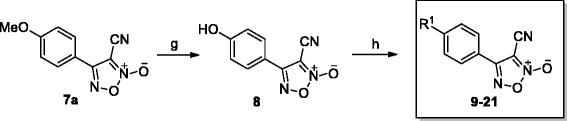
Synthesis of compounds **8–21**. *Reagents and conditions:* (g) DL-methionine, methanesulfonic acid, N_2_, 70 °C, 2 h, 53 %; (h) bromides, Cs_2_CO_3_, DMF, 30–60 °C, 40–60 %

Compounds **4a**–**4c**, **7a**–**7c** and **8** were prepared using a protocol similar to that reported by the Maloney group (Scheme 1) [15]. To make compounds **9**–**21** (Scheme 2), compound **8** was coupled with various bromides in the presence of Cs_2_CO_3_, with yields of 40–60 %. For compound **22**, compound **7b** was treated with N-bromosuccinimide (NBS) and azodiisobutyronitrile (AIBN) in CCl_4_, obtaining a yield of 54 %. Then, compound **22** was coupled with various aromatic alcohols, using Cs_2_CO_3_ as the base in DMF, to give compounds **23**–**30**, the yields of which varied from 25 to 45 %. The procedures for the preparation of compounds are detailed in Additional file 1.

### Oxadiazole-2-oxides inhibition of recombinant SjTGR

The purified recombinant SjTGR proteins were produced according to the method described by Song *et al.* [18]. The inhibition of TrxR activity by the recombinant SjTGR was performed as reported by Rai *et al.* [15]. Briefly, 50 nM SjTGR and 1 mM NADPH were incubated with different concentrations of oxadiazole-2-oxides (2, 4, 6, 10, 20 μM) for 15 min at room temperature. Then, potassium phosphate buffer (pH 7.4) containing 10 mM EDTA, 100 μM NADPH, and 3 mM DTNB was added to start the reaction. The increase of absorbance at 412 nm was monitored during the first 2 min. All assays were done in triplicate. Assays with no inhibition were performed as the control treatment. The 50 % inhibitory concentration (IC_50_) values of the oxadiazole-2-oxides were calculated by curve fitting, using SPSS 13.0 statistical software.

### Detection of nitric oxide (NO) release

The release of nitric oxide (NO) was measured as previously reported [16]. Briefly, 15 nM SjTGR, with or without 100 μM NADPH or 5 mM cysteine, was incubated at room temperature with 10 μM of the oxadiazole-2-oxides for 50 min. The NO release was detected according to the instructions of the Nitric Oxide Assay Kit, with the absorbance being measured at 540 nm. The NO release of sodium nitrite, as a standard substance, was also measured. The values of NO release were calculated on the basis of the regression line plotted for the NO release from sodium nitrite. Each reaction was done in triplicate and the data presented are the average of three independent experiments.

Healthy *S. japonica* adult worms were collected as described above and homogenized in 0.1 M potassium phosphate (pH 7.4). The homogenates were centrifuged at 20,000 *g* for 1 h at 4 °C and the supernatants were assayed for NO release. In addition, the worm homogenates (100 μg) were incubated with 10 μM oxadiazole-2-oxides for 50 min at room temperature and the production of NO was detected as above. Each reaction was done in triplicate.

### Cytotoxicity assay of oxadiazole-2-oxides on Hela cells

Cytotoxicity assays were performed using Hela cells. Cells in the exponential growth phase were collected by centrifugation and a concentration of 4–5 × 10^4^/mL was created. 100 μL of the cell suspension was added to each well of a 96-well plate and cultured overnight at 37 °C in a humid atmosphere with 5 % CO_2_. Oxadiazole-2-oxides (at 10, 20, 40, 80 and 160 μM) were added. Each concentration was assayed in triplicate, and control wells (no cells and no drugs) were assayed at the same time. The “no cell” control wells only contained RPMI 1640 medium, MTT, DMSO, and oxadiazole-2-oxides. The “no drug” control wells contained the Hela cells, RPMI 1640 medium, MTT and DMSO. The cells were cultured for 48 h at 37 °C in a humid atmosphere with 5 % CO_2_. Then, 20 μL of 5 mg/mL MTT was added to each well and the culture conditions were maintained for 4 h. After this point, the medium was removed and 150 μL DMSO was added into each well to dissolve any crystallisation. The value of absorbance at 570 nm of each well was measured (at 570 nm) with a microplate reader to assess the toxic effect of the oxadiazole-2-oxides on the vertebrate cells.

### Toxicity of oxadiazole-2-oxides on juvenile and adult *S. japonica in vitro*

Oxadiazole-2-oxides (10 μmol) were dissolved in 1 mL DMSO to prepare stock solutions, which were kept cold and in the dark. Working solutions were prepared prior to use at the appropriate dilutions, using RPMI 1640 medium.

20–30 juvenile worms were transferred into each well of a 24-well culture plate, containing 2 mL RPMI 1640 medium, 20 mM HEPES, 100 IU/mL penicillin, 100 mg/mL streptomycin and 10 % fetal bovine serum. Then, oxadiazole-2-oxides (2, 5, 10, and 50 μM), diluted with the RPMI 1640 medium, were added to their allocated wells. The worms in the negative control wells were treated with equal volumes of RPMI 1640 or DMSO and worms treated with AS were observed as positive controls. The juvenile worms’ survival rates were observed under an inverted phase contrast microscope (Leica, Wetzlar, Germany) at 24, 48 and 72 h. Parasite death was defined as an unclear internal structure of the juvenile worms, with incomplete tegument and contents overflowing radially, or unclear internal structure with complete tegument, but having no motor activity during 1 min of continuous observation [23]. All assays were repeated three times.

Adult worms were cultured in a 24-well culture plate for 30–60 min at 37 °C, in a humid atmosphere with 5 % CO_2_. Then, oxadiazole-2-oxides at different concentrations (10, 25, 50, 100 μM) were added. Equal volumes of RPMI 1640 or DMSO were applied as negative controls, while PZQ was applied as a positive control. The adult worm mobility and parasite survival rates were monitored using an inverted phase contrast microscope, at 24, 48 and 72 h. Parasite death was defined as there being no motor activity during 2 min of continuous observation, and morphological and tegumental alterations [24]. All assays were repeated three times.

To assess the importance of NO production, the potassium salt of carboxy-PTIO, a NO scavenger, was dissolved in water and 100 mM was incubated with freshly perfused adult *S. japonica* in the presence or absence of 10 mM oxadiazole-2-oxides. The worms’ mobility, tegumental alterations and parasite survival were monitored using an inverted microscope. The experiment was done in triplicate.

### Oxadiazole-2-oxides treating *S. japonica in vivo*

The abdominal skin of each mouse (ICR, female, 22–24 g) was exposed to 20 *S. japonica* cercariae (as described above). The mice were intraperitoneally injected with 10 mg/kg oxadiazole-2-oxides daily for 5 days, beginning either 7 or 35 days post-infection. Control mice were treated with 10 mg/kg of the carrier medium. All mice were sacrificed 42 days post-infection. The worm burden of each mouse was confirmed by counting the number of worms observed from a portal vein perfusion. The mouse livers were digested overnight with 5 % potassium hydroxyl solution at 37 °C and the eggs in the liver tissue were counted under an inverted phase contrast microscope. The worm and egg reduction rates were calculated using the following formulas [18]:

$$ \mathrm{Worm}\ \mathrm{reduction}\ \mathrm{rate}\ \left(\%\right)=\left(\mathrm{mean}\ \mathrm{number}\ \mathrm{of}\ \mathrm{adult}\ \mathrm{worms}\ \mathrm{per}\ \mathrm{mouse}\ \mathrm{in}\ \mathrm{control}\ \mathrm{group}-\mathrm{mean}\ \mathrm{number}\ \mathrm{of}\ \mathrm{adult}\ \mathrm{worms}\ \mathrm{per}\ \mathrm{mouse}\ \mathrm{in}\ \mathrm{experimental}\ \mathrm{group}\right)/\mathrm{mean}\ \mathrm{number}\ \mathrm{of}\ \mathrm{adult}\ \mathrm{worms}\ \mathrm{per}\ \mathrm{mouse}\ \mathrm{in}\ \mathrm{control}\ \mathrm{group}. $$

$$ \mathrm{Egg}\ \mathrm{reduction}\ \mathrm{rate}\ \left(\%\right)=\left(\mathrm{mean}\ \mathrm{number}\ \mathrm{of}\ \mathrm{eggs}\ \mathrm{per}\ \mathrm{mouse}\ \mathrm{in}\ \mathrm{control}\ \mathrm{group}-\mathrm{mean}\ \mathrm{number}\ \mathrm{of}\ \mathrm{eggs}\ \mathrm{per}\ \mathrm{mouse}\ \mathrm{in}\ \mathrm{experimental}\ \mathrm{group}\right)/\mathrm{mean}\ \mathrm{number}\ \mathrm{of}\ \mathrm{eggs}\ \mathrm{per}\ \mathrm{mouse}\ \mathrm{in}\ \mathrm{control}\ \mathrm{group}. $$

### Statistical analysis

All data are given as the mean ± standard deviation (SD). The worm and egg burden numbers after treatment with oxadiazole-2-oxides were statistically compared with the control, AS or PZQ groups using two-tailed Student’s t-tests. Differences in NO release from the oxadiazole-2-oxides compared with control were similarly assessed. Cell survival rates after exposure to oxadiazole-2-oxides and PZQ, and worm survival rates after exposure to oxadiazole-2-oxides, with or without carboxy-PTIO, were determined using chi-square tests. SPSS 13.0 was used for the statistical analyses. Differences between mean values were considered to be significant at the level of 5 %.

### Ethical approval

Ethical approval for the work performed was obtained from the Institutional Review Board (IRB00004221) of Jiangsu Institute of Parasitic Diseases, Wuxi, China (Permit Number: JIPDAERP20131120). All animals were housed in facilities and provided food and water conventionally. The infection, intraperitoneal injections and sacrifices (by carbon dioxide asphyxiation) were carried out according to recommendations in the Guidelines for the Care and Use of Laboratory Animals of the Ministry of Science and Technology of People’s Republic of China ([2006]398). Suffering was minimised in accordance with the guidelines.

## Results

### Syntheses of oxadiazole-2-oxides and their inhibitory activities against SjTGR

**Scheme 3:**
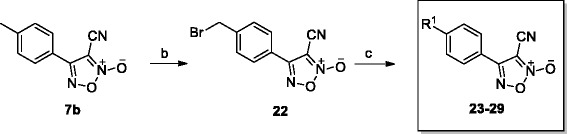
Synthesis of compounds **22–30**. *Reagents and conditions:* (b) NBS, AIBN, CCl_4_, 80 °C, overnight, 54 %; (c) aromatic alcohols, or Cs_2_CO_3_, DMF, 30–60 °C, 25–45 %

**Fig. 1:**
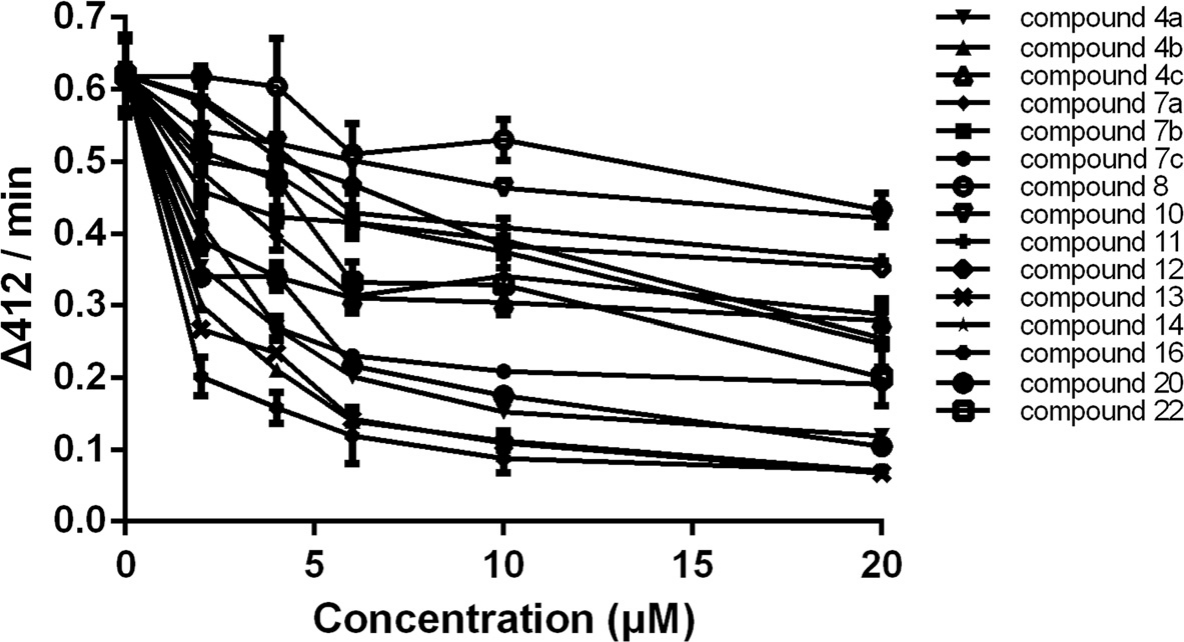
Inhibition of recombinant SjTGR by oxadiazole-2-oxides. 50 nM SjTGR and 1 mM NADPH were incubated with different concentrations of oxadiazole-2-oxides (2, 4, 6, 10, 20 μM) for 15 min at room temperature. Then, potassium phosphate buffer (pH 7.4) containing 10 mM EDTA, 100 μM NADPH, 3 mM DTNB was added to start the reaction. The increase in absorbance at 412 nm was monitored during the first 2 min. All assays were done in triplicate. Results shown are the mean ± SD (*n* = 3)

Thirty oxadiazole-2-oxides were designed and synthesised following Schemes 1, 2, 3. The structures of these oxadiazole-2-oxides are detailed in Table 1. The recombinant SjTGR was incubated with each oxadiazole-2-oxide, and their respective inhibition on the TrxR activity of SjTGR was observed. The results showed that compounds **4a**, **4b**, **4c**, **7a**, **7b**, **7c**, **8**, **11**, **12**, **13**, **14**, **16**, **20** and **22** inhibited the activity of SjTGR (Fig. 1 and Table 1), with IC_50_ values between 0.5 and 37.4 μM. Compounds **9**, **10**, **15**, **17**, **18**, **19**, **21**, **23**, **24**, **25**, **26**, **27**, **28** and **29** did not inhibit the activity of SjTGR (Table 1).

**Table 1:**
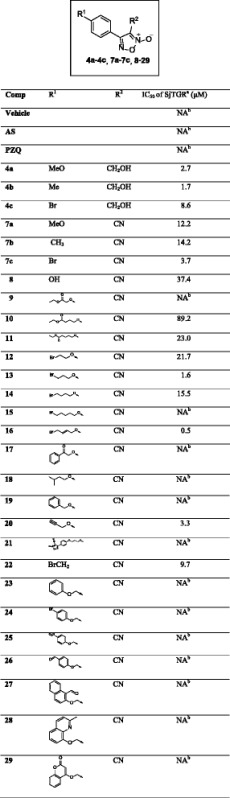
Structures of the oxadiazole-2-oxides and their inhibitory activities against SjTGR

^a^ SjTGR enzymatic assay

^b^ No activity

### NO production of oxadiazole-2-oxides *in vitro*

**Fig. 2:**
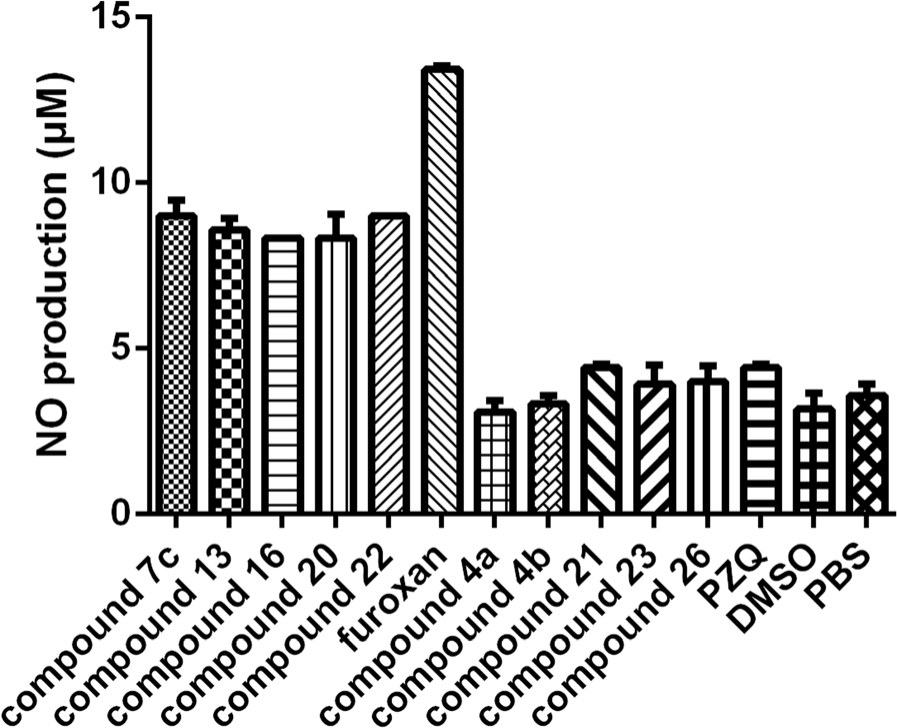
Nitrogen orxide (NO) release from oxadiazole-2-oxides incubated *in vitro.* 15 nM SjTGR, 100 μM NADPH and 5 mM cysteine were incubated with 10 μM oxadiazole-2-oxides for 50 min at room temperature. The nitric oxide (NO) released was detected according to the NO Assay Kit (Beyotime Biotechnology Corporation). The NO release levels were calculated based on the regression line of sodium nitrite (standard). Each reaction was done in triplicate. Results shown are the mean ± SD (*n* = 3)

When the oxadiazole-2-oxides were incubated with cysteine at room temperature, compounds **7c**, **13**, **16**, **20**, and **22** released 9.0 ± 0.5, 8.6 ± 0.4, 8.3 ± 0.0, 8.8 ± 0.7, and 9.0 ± 0.1 μM NO, respectively. The PBS and DMSO control wells produced 3.6 ± 0.4 and 3.2 ± 0.5 μM NO, respectively (Fig. 2). The difference in NO production between the above five compounds and DMSO was significant (*P* < 0.05). The other oxadiazole-2-oxides produced similar NO levels to the PBS and DMSO control wells (Fig. 2; *P* > 0.05). Incubation of the oxadiazole-2-oxides with SjTGR + NADPH, SjTGR alone or worm homogenate supernatant, also released similar NO to the PBS and DMSO controls (*P* > 0.05; Additional file 2: Table S1).

### Cytotoxicity of oxadiazole-2-oxides on Hela cells

The cytotoxicity assessment of the oxadiazole-2-oxides on the Hela cells was observed using the MTT method. The viability of the Hela cells immersed in 80 μM of PZQ for 72 h was 76 %, while those in 80 μM of compounds **4a**, **4b**, **7c**, **13**, **16**, **20** after 72 h were 91 %, 96 %, 66 %, 80 %, 89 % and 87 % viable, respectively. Except for compound **7c**, the toxicity of compounds **13**, **4a**, **4b**, **20**, and **16** to the Hela cells was similar to, or less than that of PZQ. The differences in toxicity were not significant, between the oxadiazole-2-oxides and PZQ (*P* > 0.05).

### Toxicity of oxadiazole-2-oxides to cultured juvenile and adult *S. japonica*

**Fig. 3:**
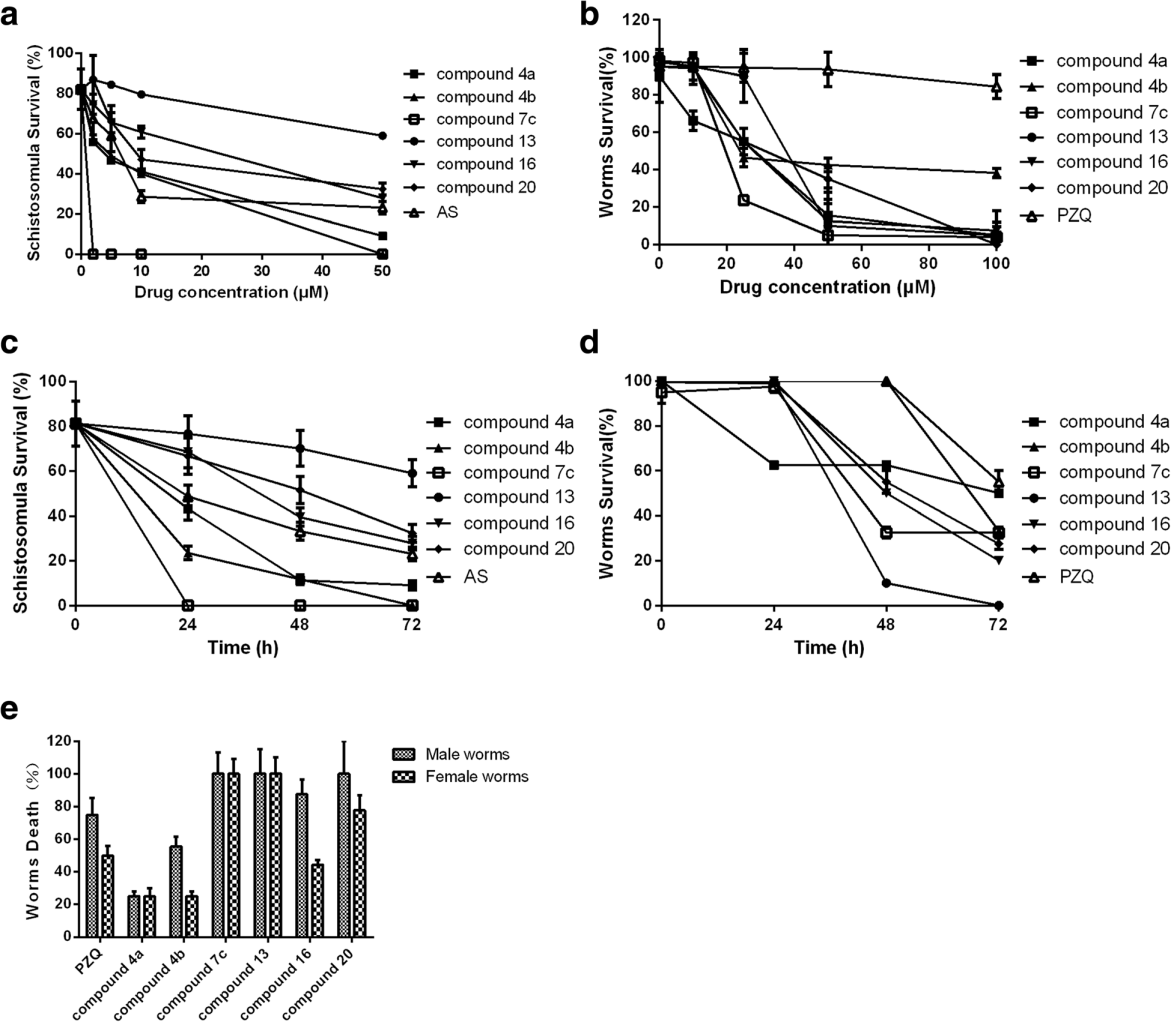
Effects of oxadiazole-2-oxides on mortality of juvenile and adult *S. japonica in vitro.* Oxadiazole-2-oxides were applied at different concentrations and mortality was measured in the juvenile worms after 72 h (**a**) and adult worms after 24 h (**b**). In **c**, the mortality of juvenile *S. japonica* caused by the oxadiazole-2-oxides (50 μM) at different exposure times is shown. In **d** the mortality of adult *S. japonica* caused by the oxadiazole-2-oxides (10 μM) at different exposure times is shown. In **e**, the mortality of male and female adult specimens of *S. japonica* exposed to 8 μM of oxadiazole-2-oxides after 120 h is shown

**Fig. 4:**
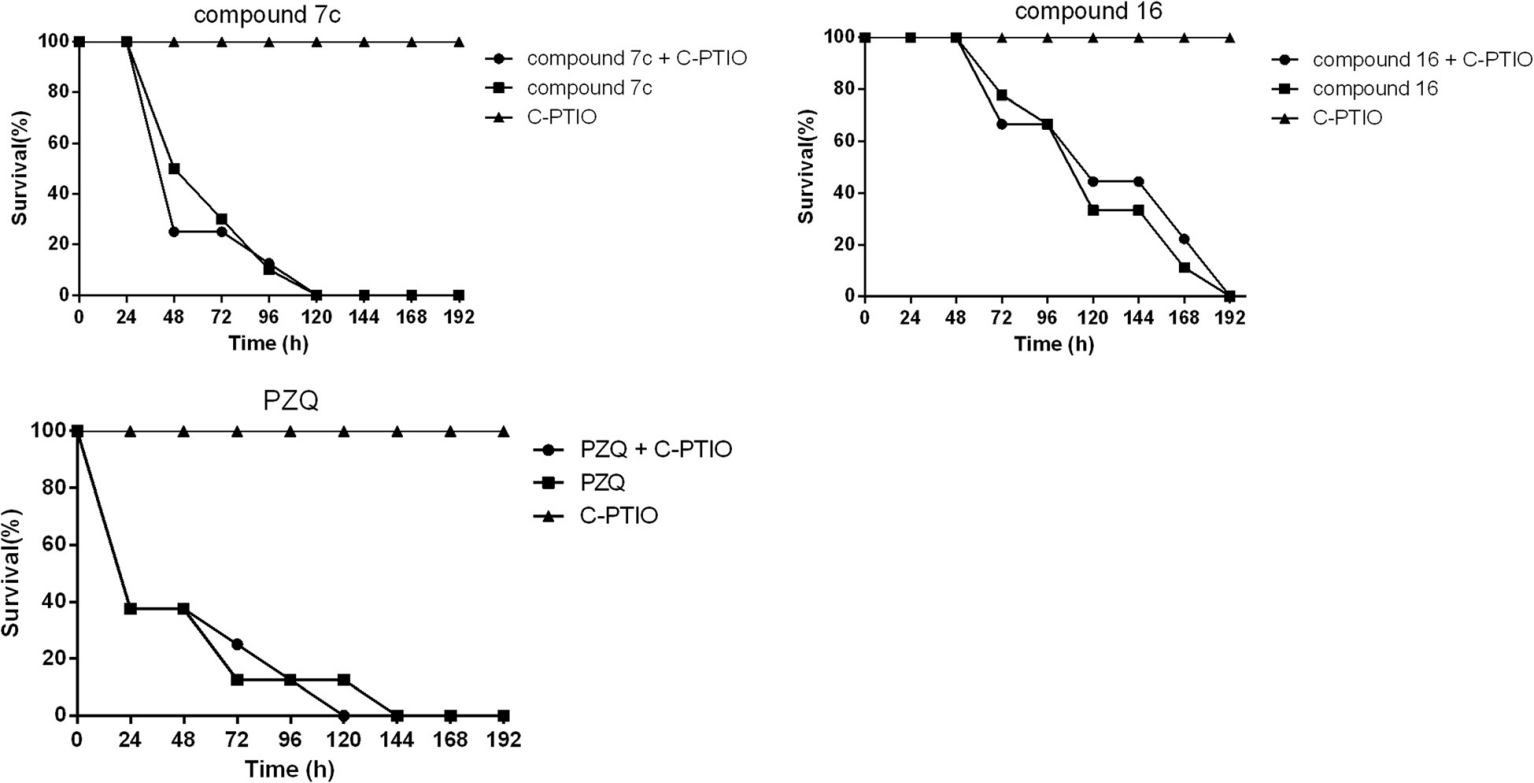
Activity of oxadiazole-2-oxides against adult *S. japonica in vitro*, with and without carboxy-PTIO. Carboxy-PTIO was incubated with freshly perfused adult *S. japonica* at 100 mM in the presence and absence of 10 mM oxadiazole-2-oxides. The worms’ mobility, tegumental alterations and survival rates were monitored under an inverted microscope. The experiment was done in triplicate. The percentage of adult worms that survived are shown (mean ± SD; *n* = 3)

The survival of juvenile and adult *S. japonica*, cultured in a medium, containing different concentrations of oxadiazole-2-oxides showed that, compared to AS – AS at 50 μM resulted in the death of 51.3 ± 5.1 % juvenile worms after 24 h – compounds **4b**, **7c**, **9**, **22** and **23** at 50 μM increased mortality, to more than 70.0 % after 24 h. In contrast, compounds **4c**, **7a**, **8**, **12**, **13**, **16**, **17**, **21** and **28** (at 50 μM) did not affect the juvenile worms, with less than 30.0 % mortality after 24 h. Compared to PZQ, which, at 25 μM resulted in the death of 25.0 ± 7.8 % adult worms after 24 h, compounds **7a**, **7b**, **7c**, **12**, **14**, **18**, **19**, **22**, **23**, **24**, **26** and **28** at 25 μM increased mortality, to more than 75.0 %. Compounds **17** and **29** did not cause death in the adult worms after 24 h, even at the maximum concentration tested (100 μM). Interestingly, we found that compounds **4a**, **7c**, **13**, **16** and **20** displayed killing activities to both the juvenile and adult worms of *S. japonica*. At concentrations of 50 μM, compounds **7c**, **13** and **16** resulted in 100.0 ± 0.0 % mortality of adult worms within 24 h, and 100.0 ± 0.0 %, 41.0 ± 6.3 % and 72.1 ± 3.7 % mortality of juvenile worms after 72 h, respectively. 100 μM of compounds **4a**, **20** caused 100.0 ± 0.0 % of the adult worms to die, and 90.0 ± 2.8 % and 67.6 ± 4.5 % of the juvenile worms died in 72 h, respectively. 100 μM of compound **4b** resulted in 57.1 ± 2.1 % adult worm death at 24 h, 100.0 ± 0.0 % adult worm mortality after 48 h, and 100.0 ± 0.0 % juvenile mortality at 72 h. The killing activities against the juvenile and adult worms of these 7 compounds increased with concentration and time (Additional file 3: Tables S2 and Additional file 4: Table S3, Fig. 3a, b, c, d). There was no statistically significant difference in mortality due to the sex of the worms, among the seven compounds (Fig. 3e). When a NO scavenger (carboxy-PTIO) was added to the culture of adult worms *in vitro*, with compounds **7c** and **16**, there were no differences in the killing activities of the compounds (with or without carboxy-PTIO; *P* > 0.05) (Fig. 4).

### The treatment effect of oxadiazole-2-oxides *in vivo*

The results of treating the mice with the oxadiazole-2-oxides *in vivo* showed that compounds **4a**, **4b**, **7c, 13**, **16** and **20** caused 87.7 %, 83.1 %, 87.1 %, 84.6 %, 90.8 % and 69.5 % mortality in the adult worms, respectively; and the eggs were reduced by 47.0 %, 0 %, 52.0 %, 52.6 %, 49.5 % and 3.3 %, respectively (when the mice were treated at the juvenile worm stage). There was a significant rise in the adult worm reduction rate of compound **16**, compared to the AS group (78.5 % in worm burdens, *P* < 0.05) (Table 2). Compounds **4a** and **16** resulted in 66.7 % and 69.4 % reductions in the worms, respectively, and 3.8 % and 20.0 % reduction in the eggs, respectively (when the mice were treated at the adult worm stage). The treatment effect of compounds **4a** and **16** were similar to that of PZQ (worm reduction rate was 72.3 %; *P* > 0.05). The other novel oxadiazole-2-oxides tested appeared to not affect the adult worms (Table 3).

**Table 2:** Treatment effects of oxadiazole-2-oxides on juvenile *S. japonicum* in mice

Groups	Mice No.	Number of worms (Mean ± SD)	Worm reduction rate (%)	Number of eggs per gram of liver (Mean ± SD)	Egg reduction rate (%)
Control	7	10.8 ± 0.8		8,032.9 ± 2,192.1	
Compound 4a	4	1.3 ± 0.8 (*P* > 0.05^**^)	87.7 (*P* < 0.01^*^)	4,258.9 ± 1,910.0 (*P* > 0.05^**^)	47.0 (*P* < 0.05^*^)
Compound 4b	2	1.8 ± 1.5 (*P* > 0.05^**^)	83.1 (*P* < 0.01^*^)	8,669.2 ± 2,255.8 (*P* < 0.05^**^)	–
Compound 7c	2	1.4 ± 0.8 (*P* > 0.05^**^)	87.1 (*P* < 0.01^*^)	3,859.4 ± 3,749.4 (*P* > 0.05^**^)	52.0 ( *P* > 0.05^*^)
Compound 13	2	1.7 ± 0.7 (*P* > 0.05^**^)	84.6 (*P* < 0.01^*^)	3,808.9 ± 1,288.9 (*P* > 0.05^**^)	52.6 (*P* < 0.05^*^)
Compound 16	5	1.0 ± 0.5 (*P* > 0.05^**^)	90.8 (*P* < 0.01^*^)	4,054.5 ± 1,322.1 (*P* > 0.05^**^)	49.5 (*P* < 0.01^*^)
Compound 20	6	3.0 ± 0.9 (*P* < 0.05^**^)	69.5 (*P* < 0.01^*^)	7,767.5 ± 2,676.6 (*P* < 0.05^**^)	3.3 (*P* > 0.05^*^)
AS	4	2.3 ± 1.0	78.5 (*P* < 0.01^*^)	2,932.2 ± 1,149.3	63.5 (*P* < 0.01^*^)

^*^Effect of compound versus control analysed by Student’s t-test; ^**^Effect of compound versus AS analysed by Student’s t-test

**Table 3:** Treatment effects of oxadiazole-2-oxides on adult *S. japonicum* in mice

Groups	Mice No.	Number of worms (Mean ± SD)	Worm reduction rate (%)	Number of eggs per gram of liver (Mean ± SD)	Egg reduction rate (%)
Control	7	12.0 ± 2.1		8032.9 ± 2192.1	
Compound 4a	4	4.0 ± 2.5 (*P* > 0.05^**^)	66.7 (*P* < 0.01^*^)	7730.8 ± 1457.8 (*P* < 0.05^**^)	3.8 (*P* > 0.05^*^)
Compound 4b	4	9.0 ± 3.8 (*P* < 0.05^**^)	25.0 (*P* > 0.05^*^)	7007.8 ± 2420.2 (*P* < 0.05^**^)	12.8 (*P* > 0.05^*^)
Compound 7c	6	12.0 ± 3.9 (*P* < 0.05^**^)	–	7213.0 ± 2658.0 (*P* < 0.05^**^)	10.2 (*P* > 0.05^*^)
Compound 13	5	8.6 ± 2.4 (*P* < 0.05^**^)	28.3 (*P* < 0.05^*^)	8201.4 ± 2066.0 (*P* < 0.05^**^)	–
Compound 16	5	3.7 ± 1.4 (*P* > 0.05^**^)	69.4 (*P* < 0.01^*^)	6424.1 ± 1193.4 (*P* < 0.05^**^)	20.0 (*P* > 0.05^*^)
Compound 20	6	4.7 ± 1.1 (*P* > 0.05^**^)	61.1 (*P* < 0.01^*^)	10094.7 ± 1691.0 (*P* < 0.05^**^)	–
PZQ	6	3.3 ± 1.7	72.3 (*P* < 0.01^*^)	3721.5 ± 1600.6	53.7 (*P* < 0.01^*^)

^*^ Effect of compound versus control analysed by Student’s t-test; ^**^Effect of compound versus PZQ analysed by Student’s t-test

## Discussion

For almost three decades, PZQ has been the only drug recommended by the World Health Organization to treat and control schistosomiasis, through mass drug administration (MDA) programs [25]. No vaccine has ever been developed [25]. PZQ does not guard against reinfection and is not effective against juvenile schistosomes. Furthermore, schistosome phenotypes are emerging that are resistant to PZQ chemotherapy, and PZQ has a limited effect on developed liver and spleen lesions [4, 26]. Although these issues have not led to treatment failures or the necessity to change control measures to date, it is imperative to develop new antischisosomal agents in anticipation of the potential threat.

Oxadiazole-2-oxides have been identified to inhibit the activity of TGR and kill *S. mansoni* worms during the *in vivo* treatment of schistosome-infected mice, which led to more than 90 % reduction in the worm burdens [15, 16]. However, there has been little attention on the effect of the oxadiazole-2-oxides against *S. japonica*, a helminth endemic to Asia. We designed and synthesised 30 oxadiazole-2-oxides, on the basis of Schemes 1, 2, 3, and studied their killing activities against juvenile and adult *S. japonica*. The mechanisms of action of the oxadiazole-2-oxides were also explored.

The *in vitro* antischistosomal activity on juvenile and adult *S. japonica*, as well as their inhibitory activity against SjTGR (assessed using our previously reported protocol [18]) showed (Table 1 and Additional file 3: Tables S2 and Additional file 4: Table S3) that compounds **7a–c** and **8** had the same structures as those described in the work with *S. mansoni* [15]. These compounds had good worm killing activity, resulting in 100.0 ± 0.0 %, 83.3 ± 4.7 %, 70.0 ± 7.1 %, and 71.4 ± 0.9 % mortality in the adult worms after 72 h, at concentrations of 10 μM, respectively; these mortality levels are similar to the data (100.0 %, 70.0 %, 100.0 %, and 100.0 %, respectively) obtained using *S. mansoni* [15]. These data show that the oxadiazole-2-oxides may have the same pharmacology in both schistosome species.

Comparing compounds **4a–4c** and **7a–7c**, the cyano group in compound **7c** replaced the hydroxyl group in compound **4c**, and at 100 μM, compound **4c** caused 57.1 ± 2.3 % mortality while compound **7c** caused 100.0 ± 0.0 % mortality in the adult worms. When the hydroxyl group in compound **4a** was changed into cyano group of compound **7a**, the activity against SjTGR slightly weakened (the IC_50_ values were 2.7 μM and 12.2 μM, respectively), but the worm killing activity of **7a** was markedly enhanced. In contrast, compound **4b** had both better activity against SjTGR and better worm killing activity, than **7b**. In compounds **9**–**11** the number of CH_2_ groups between the O atom and ester group increased, the corresponding SjTGR inhibitory effects increased, but the worm killing activity did not. Interestingly, compound **9**, which had no SjTGR inhibitory activity, caused the best worm mortality of the three compounds. Among compounds **12**–**16**, compounds **12**, **13** and **14** caused moderate SjTGR inhibition and had good worm killing activities. Compound **15** did not inhibit SjTGR and exhibited similar worm killing activity at 10 μM to compounds **12**, **13** and **14**. Changing the C–C single bond (compound **15**) into a double bond (C = C; compound **16**), inhibited SjTGR at sub-micromolar concentrations (IC_50_ = 0.54 μM), but there was no change in activity against the parasite.

As for compounds **17**–**21**, compound **18**, with an isopentyloxy group, did not inhibit SjTGR, while compound **20** with a prop-2-yn-1-yloxy group inhibited SjTGR at low micromolar concentrations; these two compounds both killed 100.0 ± 0.0 % of worms in 72 h, at concentrations of 25 μM. The symmetrical compound **21** did not inhibit SjTGR and exhibited slightly poorer worm killing activity than compound **13**. Interestingly, compound **19**, with a benzyloxy group, was the best at killing worms among all the compounds assessed. However, with a carbonyl group inserted between the phenyl group and carbon atom, compound **17** was poor at killing worms.

Among compounds **22**–**29**, those with bulkier aromatic groups (compounds **27**, **28** and **29**) caused low mortality rates, compared with compound **19**, which had a smaller phenyl group. In addition, compounds without substitution on the phenyl group (compounds **19** and **23**) were better at killing the worms than those with substitution (compounds **24**, **25** and **26**). Except for compounds **25** and **29**, the other oxadiazole-2-oxides caused higher worm mortality rates than PZQ. Compound **22** moderately inhibited SjTGR, and had a good worm killing activity; this indicates the worm killing activities of the oxadiazole-2-oxides were not directly correlated to their SjTGR inhibitory activities.

The results of the mice experiments showed large and highly significant reductions in worm burdens by treating the mice at juvenile worm stage with compounds **4a**, **4b**, **7c**, **13** and **16**, compared to AS. The treatment effects of compounds **4a** and **16** were almost the same as PZQ, at the adult worm stage. These results generally agree with Rai *et al.* [15], who reported that oxadiazole-2-oxide derivatives nearly cured *S. mansoni*-infected mice, with a 90 % reduction in the worm burdens. However, there was a contradiction between the worm and egg reduction rates in the PZQ and oxadiazole-2-oxides treatments for the mice; compounds **4a** and **16** seemed to eliminate the adult parasites essentially to the same extent as PZQ, but had less effect on the egg burden than PZQ. The main reason was that the reductions in the female worms due to treatment with compounds **4a** and **16** were 36.8 % and 44.7 %, which was less than the reduction in female worms due to PZQ treatment (60.5 %) (data not shown). This structure activity relationship (SAR) could have important implications for further drug development of the oxadiazole-2-oxides against schistosomiasis.

The oxadiazole-2-oxide core is a class of NO donating compounds [27]. NO is a free radical and is a well-studied physiological signaling agent; it is best known for its ability to relax smooth muscle tissue, resulting in vasodilatation and increased blood flow at low concentrations [28]. However, elevated levels of NO can be toxic, which can kill normal cells, tumuor cells, bacteria and parasites [29–31]. Rai *et al.* [15] proposed that oxadiazole-2-oxides kill *S. mansoni* due to the free thiols of TGR, which can attack the oxadiazole-oxide, affecting its molecular arrangement and releasing of NO; they supposed that the concomitant inhibition of TGR activity would result in the accumulation of reactive oxygen species and increase oxidative stress. However, our NO release and scavenger experiment showed that when compounds **7c**, **13**, **16**, **20** and **22**, and furoxan with a cyano group, were incubated with cysteine, the release of NO increased. When the cyano group of compounds **4a** and **4b** were replaced with a hydroxyl group, no NO release was observed. These results show that the release of NO was related to the cyano group. However compounds **21**, **23** and **26** had a cyano group, but did not release NO. Compared with the structure of compounds **7c**, **13**, **16**, **20** and **22**, this may be related to the hydrogen, which was replaced by a benzene ring, influencing the spatial structure of the compounds. Therefore, the release of NO may be relevant to the position of the cyano group in the compound. Compounds **4a**, **4b**, **7c**, **13**, **16**, **20** and **22** released NO and the values of their activity against SjTGR were all under 10 μM. Compounds **19**, **21** and **26** did not release NO and neither did they inhibit the SjTGR activity. It appears that the release of NO may inhibit the activity of SjTGR. However, compounds **4a** and **4b** did not release NO, but did strongly inhibit SjTGR, with IC_50_ values of 2.7 and 1.7 μM. These results indicate that oxadiazole-2-oxides have other mechanisms against SjTGR activity.

In addition, when the oxadiazole-2-oxides were incubated with SjTGR or worm homogenate for 50 min at room temperature, no NO release was observed. This illustrates that the relationship between SjTGR inhibition and the release of NO by oxadiazole-2-oxides needs further investigation. Even though compounds **23** and **26** did not release NO they had good activity against the worms *in vitro*; the NO clear experiment showed that there was no difference in the killing activity of the oxiadiazole-2-oxides against the worms *in vitro,* with or without carboxy-PTIO. This shows that the effects of the drugs against the worms were not correlated with the NO release from the oxiadiazole-2-oxides, indicating that there are other mechanisms, besides NO, that led to mortality of the parasites. That is, the oxadiazole-2-oxides synthesised in this study may have another target, in addition to SjTGR, through which they kill *S. japonica*.

Our study shows that there were no obvious correlations among the activity of oxadiazole-2-oxides against *S. japonicum* worms, SjTGR inhibition and the production of NO. One reason for this could be as suggested by Rai *et al*. [15]; worm death may not occur until the GSH/GSSG ratio reaches a critical point and the overall worm redox balance is unrecoverable, making it difficult to derive correlations between mortality in the worms and TGR inhibition below a certain level. In addition, Treger *et al.* [32] reported that oxadiazole-2-oxides kill *Ancylostoma ceylanicum* and proved that their target was not glutathione reductase. Besides these reasons, several studies [33–36] have shown that oxadiazole-2-oxides have many functions, which have been used in new drug developments against bacteria, fungi, viruses, worms, tumors, syncopes and immune suppressors. This indicates that oxadiazole-2-oxides may have a lot of function targets. Our study has shown that oxadiazole-2-oxides may have other targets and mechanisms, in addition to SjTGR and its inhibition, within *S. japonicum*.

## Conclusions

In this study, a series of novel oxadiazole-2-oxides were designed and synthetised. Some of them killed juvenile and adult *S. japonica* effectively. The antischistosomal activity of these compounds was related to their structure, but not positively correlated to the inhibition of SjTGR or NO release. These oxadiazole-2-oxides may serve as the leads to develop novel antischistsosomal drugs and explore potential molecular targets further.

## Additional files

Additional file 1:
**Procedures for the preparation of compounds.** (DOCX 36 kb) Additional file 2: Table S1. Nitric Oxide (NO) production of oxadiazole-2-oxides with SjTGR+NADPH or with SjTGR alone, or with worm homogenate supernatant. (DOCX 17 kb) Additional file 3: Table S2. Killing activity on juvenile *S. japonicum in vitro* by oxadiazole-2-oxides. (DOCX 27 kb) Additional file 4: Table S3. Killing activity on adult *S. japonicum in vitro* by oxadiazole-2-oxides. (DOCX 23 kb)

## Abbreviations

TGR: thioredoxin glutathione reductase
SjTGR: *S. japonicum* thioredoxin glutathione reductase
DTNB: 5, 5’-Dithiobis-(2-nitrobenzoic acid)
NADPH: β-Nicotinamide adenine dinucleotide 2'-phosphate reduced tetrasodium salt
MTT: 3-(4, 5-dimethyl-2-thiazolyl)-2, 5-diphenyl-2-H-tetrazolium bromide
DMSO: dimethyl sulphoxide
AS: artemisinin
PZQ: praziquantel
carboxy; PTIO: Nitric Oxide Assay Kit and 2 - (4 - carboxyphenyl) - 4, 4, 5, 5 - tetramethylimidazoline −1- oxyl – 3 - oxide
EDTA: Ethylenediaminetetraacetic acid
HEPES: 2-[4-(2-Hydroxyethyl)-1-piperazinyl] ethanesulfonic acid
NO: nitric oxide
SD: standard deviation
SAR: structure-activity relationship
