# Supplementary material for: Oxadiazole-2-oxides may have other functional targets, in addition to SjTGR, through which they cause mortality in Schistosoma japonicum
Source: Parasit Vectors. 2016 Jan 20;9:26. doi: 10.1186/s13071-016-1301-3 (PMC4721062; doi:10.1186/s13071-016-1301-3)
Supplement: Additional file 1: — Procedures for the preparation of compounds. (DOCX 36 kb) [file 13071_2016_1301_MOESM1_ESM.docx]

**Additional file 1: Procedures for the preparation of compounds**

All chemicals were reagent grade and used as purchased. All reactions were performed under an inert atmosphere of dry argon or nitrogen using distilled dry solvent. ^1^H (400 MHz) and ^13^C (100 MHz) NMR spectra were recorded on a Bruker AVⅢ 400MHz/100MHz spectrometer. The chemical shifts were reported in (ppm) using the 7.26 signal of CDCl_3_ (^1^H NMR) and the 77.0 signal of CDCl_3_ (^13^C NMR) as internal standards. High-resolution MS data were obtained on a Agilent TOF G6224 mass spectrometer.

1. *The preparation of compounds* ***4a-4c and 7a-7c***

To a solution containing compound **1a** (10 mmol) in absolute ethanol (50 mL) was added Conc H_2_SO_4_ (50 μL) and the reaction was refluxed overnight. After completion of the reaction, the solvent was removed under diminished pressure and the residue was re-dissolved in ethyl acetate. The ethyl acetate layer was washed successively with saturated NaHCO_3_, water, brine then dried (Na2SO4) and concentrated under diminished pressure to give the pure product without need of further purification for next step.

To a suspension of **2a** (10 mmol) in toluene (25 mL) at –78 ^o^C was added dropwise DIBAL (22 mmol, 1.0 M solution in toluene) over 45 min. The reaction mixture was allowed to warm to room temperature over 2 h and then stirred this temperature for an additional hour. The reaction mixture was quenched with ice containing dilute HCl. The organic layer was separated and the aqueous layer was extracted with ethyl acetate. The combined organic layer was washed with brine and water, dried (MgSO4), filtered and concentrated under diminished pressure. The crude residue was purified on column chromatography to give compound **3a** (1.56 g, 95%).

To a solution of **3a** (1.56 g, 9.5 mmol) in glacial acetic acid (50 mL) was added sodium nitrite (0.68 g, 10mmol) portion wise over 45 min. The reaction mixture was stirred at RT 24 h. After completion of the reaction, the reaction mixture was quenched with ice water and extracted with ethyl acetate. The ethyl acetate layer was washed successively with saturated NaHCO_3_, water, brine then dried (Na_2_SO_4_) and concentrated under diminished pressure to give the crude product. The crude residue was purified on column chromatography to give compound **4a** (0.72g, 32%). ^1^HNMR (400MHz, CDCl_3_) *δ*: 7.77 (d, *J* = 8.8 Hz, 2H), 7.05 (d, *J* = 8.8 Hz, 2H), 4.74 (s, 2H), 3.89 (s, 3H).

Using the similar protocol to make the compound **4b** and **4**c.

*3-(hydroxymethyl)-4-(p-tolyl)-1, 2, 5-oxadiazole 2-oxide* (**4b**) Yield = 25%; ^1^HNMR (400MHz, CDCl_3_) *δ*: 7.70 (d, *J* = 8.0 Hz, 2H), 7.35 (d, *J* = 8.0 Hz, 2H), 4.75 (s, 2H), 2.45 (s, 3H).

*4-(4-bromophenyl)-3-(hydroxymethyl)-1, 2, 5-oxadiazole 2-oxide* (**4c**) Yield = 30%; ^1^HNMR (400MHz, CDCl_3_) *δ*: 7.74 (d, *J* = 8.8 Hz, 2H), 7.69 (d, *J* = 8.8 Hz, 2H), 4.73 (d, *J* = 5.2 Hz, 2H).

Compound **4a** (2.22g, 10 mmol) was dissolved in dichloromethane (50 mL) and treated with activated manganese dioxide (150 mmol). The reaction mixture was stirred at RT for 12 h then filtered through celite and concentrated under diminished pressure. The crude product was purified on column chromatography to give compound **5a** (2.02, 92%). compound **5a** (9.2 mmol), hydroxylamine hydrochloride (15 mmol) and sodium acetate (10 mmol) in ethanol (50 mL) was refluxed for 3h. After completion of the reaction, the solvent was removed under diminished pressure and the crude residue was purified on column chromatography to give compound **6a** (1.05g, 49%). To a solution of **6a** (1.05g, 4.5 mmol) in DMF (4 mL) was added drop wise thionyl chloride (4 mmol) at 0 ^o^C. The reaction mixture was allowed to warm to room temperature with stirring over 3 h then stirred at this temperature for an additional hour. The reaction mixture was quenched with ice and extracted with dichloromethane. The combined organic layer was successively washed with saturated NaHCO_3_, water, brine then dried (Na_2_SO_4_) and concentrated under diminished pressure to give the crude product. The crude product was purified on column chromatography to give **7a** (732mg, 75%). ^1^HNMR (400MHz, CDCl_3_) *δ*: 7.88 (d, *J* = 8.8 Hz, 2H), 7.07 (d, *J* = 8.8 Hz, 2H), 3.90 (s, 3H).

Using the similar protocol to make compound the **7b** and **7**c.

*3-cyano-4-(p-tolyl)-1, 2, 5-oxadiazole 2-oxide* (**7b**) Yield = 70%; ^1^HNMR (400MHz, CDCl_3_) *δ*: 7.75 (d, *J* = 8.0 Hz, 2H), 7.32 (d, *J* = 8.0 Hz, 2H), 2.39 (s, 3H).

*4-(4-bromophenyl)-3-cyano-1, 2, 5-oxadiazole 2-oxide* (**7c**) Yield = 73%; ^1^HNMR (400MHz, CDCl_3_) *δ*: 7.81 (d, *J* = 8.8 Hz, 2H), 7.74 (d, *J* = 8.8 Hz, 2H).

1. *The preparation of compound* ***8***

DL-Methionine (1.03g, 7mmol) was added to the mixture of the tosyl (25ml) and compound **7a** (1g, 4.6mmol) under N_2_ at 70^o^C and monitored by TLC. After 2h, the resulting reaction was brought to pH 6~7 using saturated aqueous NaHCO_3_. The reaction was extracted with ethyl acetate (3×50mL) and then washed with brine (50mL), dried over Na_2_SO_4_. The crude product was then purified by column chromatography (petroleum ether / EtOAc 10:1) to afford compound **8** (500mg, 53%). ^1^HNMR (400MHz, CDCl_3_) *δ*: 7.77 (d, *J* = 8.8 Hz, 2H), 6.95 (d, *J* = 8.8 Hz, 2H).

*3. The preparation of compound* ***22***

A mixture of compound **7b** (0.4 g, 2.0 mmol), NBS (0.39 g, 2.2 mmol) and AIBN (0.04 g, 0.22 mmol) in CCl_4_ (100 mL) was refluxed N_2_ atmosphere for 3 h. The solvent was evaporated and the residue was purified via column chromatography to afford compound **22** (0.3 g, 54 %). ^1^HNMR (400MHz, CDCl_3_) *δ*: 4.46 (s, 2H), 7.55 (d, *J* = 8.4 Hz, 2H), 7.85 (d, *J* = 8.4 Hz, 2H); HRMS (ESI) *m/z* 301.1406 [M+Na]^+^ (calcd for C_10_H_6_BrN_3_NaO_2_ 301.9536).

*4. General procedure for the preparation of derivatives* ***9****-****21***

A mixture of compound **8** (50 mg, 0.24 mmol), Cs_2_CO_3_ (96.4 mg, 0.30 mmol) and Ethyl bromoacetate (27 μL, 0.24 mmol) in DMF (2 mL) was refluxed N_2_ atmosphere for 3 hours. The reaction mixture was diluted with EtOAc (50 mL) and H_2_O (20 mL). The aqueous phase was extracted with EtOAc (40 mL). The combined organic phases were then processed in the usual way and chromatographed (5:1 petroleum ether / EtOAc ) to yield compound **9** (28 mg, 41%)*.*

*4.1. 3-cyano-4-(4-(2-ethoxy-2-oxoethoxy)phenyl)-1,2,5-oxadiazole 2-oxide* (**9**) Yield = 41%; ^1^H NMR (400 MHz, CDCl_3_) *δ*: 1.25 (t, *J* = 7.2 Hz, 3H), 4.23 (q, *J* = 7.2 Hz, 2H), 4.65 (s, 2H), 7.01 (d, *J* = 8.8 Hz, 2H), 7.82 (d, *J* = 8.8 Hz, 2H); ^13^C NMR (100 MHz, CDCl_3_) *δ*: 14.2, 61.7, 65.2, 95.5, 106.6, 115.8, 117.1, 128.5, 153.8, 161.2, 168.0; HRMS (ESI) *m/z* 290.2689 [M+H]^+^ (calcd for C_13_H_12_N_3_O_5_ 290.0771).

*4. 2*. *3-cyano-4-(4-(4-ethoxy-4-oxobutoxy)phenyl)-1,2,5-oxadiazole 2-oxide* (**10**) Yield = 45%; ^1^HNMR (400MHz, CDCl_3_) *δ*: 1.20 (t, *J* = 7.2 Hz, 3H), 2.07-2.11 (m, 2H), 2.47(t, *J* = 7.2 Hz, 2H), 4.03 (t, *J* = 7.2 Hz, 2H), 4.09 (q, *J* = 7.2 Hz, 2H), 6.98 (d, *J* = 8.8 Hz, 2H), 7.79 (d, *J* = 8.8 Hz, 2H); ^13^C NMR (100 MHz, CDCl_3_) *δ*: 14.3, 24.4, 30.6, 60.6, 67.2, 95.5, 106.7, 115.6, 116.0, 128.6, 154.0, 162.3, 173.0; HRMS (ESI) *m/z* 318.3024 [M+H]^+^ (calcd for C_15_H_16_N_3_O_5_ 318.1084).

*4. 3. 3-cyano-4-(4-((5-ethoxy-5-oxopentyl)oxy)phenyl)-1,2,5-oxadiazole 2-oxide* (**11**) Yield = 50%; ^1^HNMR (400MHz, CDCl_3_) *δ*: 1.20 (t, *J* = 7.2 Hz, 3H), 1.77-1.79 (m, 4H), 2.34 (t, *J* = 8.0 Hz, 2H), 3.99 (t, *J* = 7.0 Hz, 2H), 4.07 (q, *J* = 7.2 Hz, 2H), 6.98 (d, *J* = 8.8 Hz, 2H), 7.79 (d, *J* = 8.8 Hz, 2H); ^13^C NMR (100 MHz, CDCl_3_) *δ*: 14.3, 21.6, 28.5, 33.9, 60.4, 67.9, 95.5, 106.8, 115.6, 115.9, 128.6, 154.0, 162.5, 173.3; HRMS (ESI) *m/z* 332.2793 [M+H]^+^ (calcd for C_16_H_18_N_3_O_5_ 332.1241).

*4. 4. 4-(4-(2-bromoethoxy)phenyl)-3-cyano-1,2,5-oxadiazole 2-oxide* (**12**) Yield = 48%; ^1^HNMR (400MHz, CDCl_3_) *δ*: 3.62 (t, *J* = 6.0 Hz, 2H), 4.32 (t, *J* = 6.0 Hz, 2H), 7.72 (d, *J* = 8.8 Hz, 2H), 7.82 (d, *J* = 8.8 Hz, 2H); ^13^C NMR (100 MHz, CDCl_3_) *δ*: 29.7, 68.0, 95.5, 106.7, 115.8, 116.8, 128.7, 153.8, 161.5.

*4. 5. 4-(4-(3-bromopropoxy)phenyl)-3-cyano-1,2,5-oxadiazole 2-oxide* (**13**) Yield = 55%; ^1^HNMR (400MHz, CDCl_3_) *δ*: 2.30 (m, 2H), 3.56 (t, *J* = 6.4 Hz, 2H), 4.14 (t, *J* = 6.0 Hz, 2H), 7.01 (d, *J* = 8.8 Hz, 2H), 7.81 (d, *J* = 8.8 Hz, 2H); ^13^C NMR (100 MHz, CDCl_3_) *δ*: 24.4, 30.6, 67.2, 95.4, 106.7, 115.6, 116.0, 128.6, 154.0, 162.3; HRMS (ESI) *m/z* 346.3316 [M+Na]^+^ (calcd for C_12_H_10_BrN_3_NaO_3_ 345.9803).

*4. 6. 4-(4-(4-bromobutoxy)phenyl)-3-cyano-1,2,5-oxadiazole 2-oxide* (**14**) Yield = 60%; ^1^HNMR (400MHz, CDCl_3_) *δ*: 1.92-2.05 (m, 4H), 3.44 (t, *J* = 6.4 Hz, 2H), 4.02 (t, *J* = 6.0 Hz, 2H), 6.98 (d, *J* = 8.8 Hz, 2H), 7.80 (d, *J* =8.8 Hz, 2H); ^13^C NMR (100 MHz, CDCl_3_) *δ*: 27.7, 29.3, 33.2, 67.3, 95.5, 106.7, 115.6, 116.1, 128.6, 154.0, 162.3.

*4. 7. 4-(4-((5-bromopentyl)oxy)phenyl)-3-cyano-1,2,5-oxadiazole 2-oxide* (**15**) Yield = 54%; ^1^HNMR (400MHz, CDCl_3_) *δ*: 1.55-1.63 (m, 2H), 1.76-1.97 (m, 4H), 3.38 (t, *J* = 6.4 Hz, 2H), 3.99 (t, *J* = 6.4 Hz, 2H), 6.98 (d, *J* = 8.8 Hz, 2H), 7.79 (d, *J* = 8.8 Hz, 2H); ^13^C NMR (100 MHz, CDCl_3_) *δ*: 24.8, 28.2, 32.4, 33.5, 68.0, 95.5, 106.8, 115.6, 116.0, 128.6, 154.0, 162.5; HRMS (ESI) *m/z* 374.3634 [M+Na]^+^ (calcd for C_14_H_14_BrN_3_NaO_3_ 374.0111).

*4. 8. (E)-4-(4-((4-bromobut-2-en-1-yl)oxy)phenyl)-3-cyano-1,2,5-oxadiazole 2-oxide* (**16**) Yield = 55%; ^1^HNMR (400MHz, CDCl_3_) *δ*: 4.17 (d, *J* = 4.4 Hz, 2H), 4.57 (d, *J* = 5.2 Hz, 2H), 5.88-6.02 (m, 2H), 7.00 (d, *J* = 8.8 Hz, 2H), 7.80 (d, *J* = 8.8 Hz, 2H); ^13^C NMR (100 MHz, CDCl_3_) *δ*: 62.7, 68.2, 95.5, 106.7, 115.8, 116.2, 124.9, 128.6, 133.7, 153.9, 162.0.

*4. 9. 3-cyano-4-(4-(2-oxo-2-phenylethoxy)phenyl)-1,2,5-oxadiazole 2-oxide* (**17**) Yield = 60%; ^1^HNMR (400MHz, CDCl_3_) *δ*: 5.42 (s, 2H), 7.10 (d, *J* = 8.8 Hz, 2H), 7.54 (t, *J* = 7.6 Hz, 2H), 7.66 (t, *J* = 7.6 Hz, 1H), 7.88 (d, *J* = 8.8 Hz, 2H), 8.01(d, *J* = 8.0 Hz, 2H); ^13^C NMR (100 MHz, CDCl_3_) *δ*: 70.5, 95.5, 106.7, 115.9, 117.0, 128.1, 128.7, 129.1, 134.1, 134.3, 153.8, 161.4, 193.2; HRMS (ESI) *m/z* 344.0641 [M+Na]^+^ (calcd for C_17_H_11_N_3_NaO_4_ 344.0642).

*4. 10. 3-cyano-4-(4-(isopentyloxy)phenyl)-1,2,5-oxadiazole 2-oxide* (**18**) Yield = 46%; ^1^HNMR (400MHz, CDCl_3_) *δ*: 0.91 (d, *J* = 6.4 Hz, 6H), 1.63-1.68 (m, 2H), 1.75-1.82 (m, 1H), 4.00 (t, *J* = 6.4 Hz, 2H), 6.98 (d, *J* = 8.8 Hz, 2H) 7.79 (d, *J* = 8.8 Hz, 2H); ^13^C NMR (100 MHz, CDCl_3_) *δ*: 22.7, 25.0, 37.7, 66.9, 95.5, 106.8, 115.6, 115.7, 128.5, 154.1, 162.6; HRMS (ESI) *m/z* 344.0641 [M+Na]^+^ (calcd for C_17_H_11_N_3_NaO_4_ 344.0642).

*4. 11. 4-(4-(benzyloxy)phenyl)-3-cyano-1,2,5-oxadiazole 2-oxide* (**19**) Yield = 45%; ^1^HNMR (400MHz, CDCl_3_) *δ*: 5.16 (s, 2H), 7.14 (d, *J* = 8.8 Hz, 2H), 7.37-7.46 (m, 5H), 7.87 (d, *J* = 8.8 Hz, 2H); ^13^C NMR (100 MHz, CDCl_3_) *δ*: 70.3, 95.6, 106.7, 116.0, 116.2, 127.5, 128.4, 128.6, 128.8, 135.9, 153.9, 162.2.

*4. 12. 3-cyano-4-(4-(prop-2-yn-1-yloxy)phenyl)-1,2,5-oxadiazole 2-oxide* (**20**) Yield = 58%; ^1^HNMR (400MHz, CDCl_3_) *δ*: 2.51 (d, *J* = 2.4 Hz, 1H), 4.72 (d, *J* = 2.4 Hz, 2H), 7.09 (d, *J* = 8.8 Hz, 2H), 7.83 (d, *J* = 8.8 Hz, 2H).

*4.13. 4,4'-((propane-1,3-diylbis(oxy))bis(4,1-phenylene))bis(3-cyano-1,2,5-oxadiazole 2-oxide)* (**21)** Yield = 40%; ^1^HNMR (400MHz, CDCl_3_) *δ*: 2.30 (t, *J* = 6.0 Hz, 2H), 4.21 (t, *J* = 6.0 Hz, 4H), 7.02 (d, *J* = 8.8 Hz, 4H), 7.81 (d, *J* = 8.8 Hz, 4H); ^13^C NMR (100 MHz, CDCl_3_): *δ* 28.9, 64.5, 95.5, 106.7, 115.6, 116.2, 128.6, 153.9, 162.2; HRMS (ESI) *m/z* 469.0873 [M+Na]^+^ (calcd for C_21_H_14_N_6_NaO_6_ 469.0873).

*5. General procedure for the preparation of derivatives* ***23****-****29***

A mixture of compound **22** (50 mg, 0.18 mmol), Cs_2_CO_3_ (72 mg, 0.22 mmol) and phenol (17 mg, 0.18 mmol) in DMF (2 mL) was stirred at 40 ^o^C under N_2_ atmosphere for 3 hours. The reaction mixture was diluted with EtOAc (50 mL) and H_2_O (20 mL). The aqueous phase was extracted with EtOAc (40 mL). The combined organic phases were then processed in the usual way and chromatographed (5:1 petroleum ether / EtOAc ) to yield compound **23** (23 mg, 45%).

*5.1. 3-cyano-4-(4-(phenoxymethyl)phenyl)-1, 2, 5-oxadiazole 2-oxide* (**23**) Yield = 45%; ^1^HNMR (400 MHz, CDCl_3_) *δ*: 5.10 (s, 2H), 6.87-6.95 (m, 3H), 7.19-7.27 (m, 2H), 7.59 (d, *J* = 8.0 Hz, 2H), 7.88 (d, *J* = 8.0 Hz, 2H).

*5.2. 4-(4-((4-bromophenoxy)methyl)phenyl)-3-cyano-1,2,5-oxadiazole 2-oxide* (**24**) Yield = 25%; ^1^HNMR (400 MHz, CDCl_3_) *δ*: 5.07 (s, 2H), 6.79 (d, *J* = 8.8 Hz, 2H), 7.33 (d, *J* = 9.2 Hz, 2H), 7.57 (d, *J* = 8.0 Hz, 2H), 7.88 (d, *J* = 8.4 Hz, 2H); ^13^C NMR (100 MHz, CDCl_3_): *δ* 69.2, 95.6, 106.4, 113.7, 116.6, 123.4, 127.2, 128.2, 132.5, 141.9, 154.0, 157.4; HRMS (ESI) *m/z* 764.5737 [2M+Na]^+^ (calcd for C_32_H_20_Br_2_N_6_NaO_6_ 764.9703).

*5.3. 3-cyano-4-(4-((4-nitrophenoxy)methyl)phenyl)-1,2,5-oxadiazole 2-oxide* (**25**) Yield = 43%; ^1^HNMR (400MHz, CDCl_3_) *δ*: 5.20(s, 2H), 6.99 (d, *J* = 8.8 Hz, 2H), 7.59 (d, *J* = 8.8 Hz, 2H), 7.91 (d, *J* = 8.8 Hz, 2H), 8.7 (d, *J* = 8.8 Hz, 2H); ^13^C NMR (100 MHz, CDCl_3_): *δ* 69.5, 95.5, 106.5, 114.8, 126.1, 127.4, 127.6, 128.3, 132.6, 140.8, 153.1, 163.0.

*5.4. 3-cyano-4-(4-((4-formylphenoxy)methyl)phenyl)-1,2,5-oxadiazole 2-oxide* (**26**) Yield = 43%; ^1^HNMR (400MHz, CDCl_3_) *δ*: 5.19 (s, 2H), 7.03 (d, *J* = 8.8 Hz, 2H), 7.60 (d, *J* = 8.8 Hz, 2H), 7.81 (d, *J* = 8.8 Hz, 2H), 7.91 (d, *J* = 8.8 Hz, 2H), 9.84 (s, 1H); ^13^C NMR (100 MHz, CDCl_3_): *δ* 69.2, 95.5, 106.4, 115.1, 123.7, 127.4, 128.3, 130.6, 132.4, 141.2, 153.9, 163.1, 190.7; HRMS (ESI) *m/z* 643.2922 [2M+H]^+^ (calcd for C_34_H_23_N_6_O_8_ 643.1572).

*5.5. 3-cyano-4-(4-(((1-formylnaphthalen-2-yl)oxy)methyl)phenyl)-1,2,5-oxadiazole 2-oxide* (**27**) Yield = 36%; ^1^HNMR (400 MHz, CDCl_3_) *δ*: 5.33 (s, 2H), 7.20 (m, 1H), 7.39 (t, *J* = 7.8 Hz, 1H), 7.52 (d, *J* = 8.0 Hz, 2H), 7.58 (t, *J* = 8.0 Hz, 1H), 7.65 (d, *J* = 8.0 Hz, 2H), 7.72 (d, *J* = 8.0 Hz, 1H), 7.99 (d, *J* = 9.2 Hz, 1H) 9.20 (d, *J* = 8.8 Hz, 1H), 10.93 (s, 1H); ^13^C NMR (100 MHz, CDCl_3_): *δ* 70.4*,* 94.0, 106.4, 112.4, 113.4, 117.5, 118.5, 125.0, 125.3, 127.6, 128.3, 128.9, 130.2, 131.6, 132.7, 137.7, 141.3, 162.3, 191.5; HRMS (ESI) *m/z* 394.0797 [M+Na]^+^ (calcd for C_21_H_13_N_3_NaO_4_ 394.0798).

*5.6. 3-cyano-4-(4-(((2-methylquinolin-8-yl)oxy)methyl)phenyl)-1,2,5-oxadiazole 2-oxide* (**28**) Yield = 39%; ^1^HNMR (400 MHz, CDCl_3_) *δ*: 2.76 (s, 3H), 5.47 (s, 2H) 6.90 (d, *J* = 8.0 Hz, 1H), 7.23-7.33 (m, 3H), 7.68 (d, *J* = 8.0 Hz, 2H), 7.85 (d, *J* = 8.0 Hz, 2H), 7.97 (d, *J* = 8.4 Hz, 1H); ^13^C NMR (100 MHz, CDCl_3_): *δ* 25.6, 70.0, 95.6, 106.5, 110.7, 120.5, 122.9, 123.0, 125.7, 127.1, 127.3, 127.9, 132.4, 136.6, 142.7, 153.1, 154.1, 158.5; HRMS (ESI) *m/z* 359.1181 [M+H]^+^ (calcd for C_20_H_15_N_4_O_3_ 359.1139).

*5.7. 3-cyano-4-(4-(((2-oxo-2H-chromen-4-yl)oxy)methyl)phenyl)-1,2,5-oxadiazole 2-oxide* (**29**) Yield = 35%; ^1^HNMR (400 MHz, CDCl_3_) *δ*: 5.25 (s, 2H), 5.72 (s, 1H), 7.23-7.30 (m, 2H), 7.53 (m, 1H), 7.63 (d, *J* = 8.4 Hz, 2H), 7.82 (m, 1H), 7.95 (d, *J* = 8.4 Hz, 2H); ^13^C NMR (100 MHz, CDCl_3_): *δ* 70.0, 91.6, 95.5, 106.4, 115.4, 117.0, 123.0, 124.1, 124.3, 127.5, 128.5, 132.8, 139.4, 153.4, 153.7, 162.5, 165.0; HRMS (ESI) *m/z* 362.3267 [M+H]^+^ (calcd for C_19_H_12_N_3_O_5_ 362.0771).
