# Supplementary material for: Oxadiazole-2-oxides may have other functional targets, in addition to SjTGR, through which they cause mortality in Schistosoma japonicum
Source: Parasit Vectors. 2016 Jan 20;9:26. doi: 10.1186/s13071-016-1301-3 (PMC4721062; doi:10.1186/s13071-016-1301-3)
Supplement: Additional file 2: Table S1. — Nitric Oxide (NO) production of oxadiazole-2-oxides with SjTGR+NADPH or with SjTGR alone, or with worm homogenate supernatant. (DOCX 17 kb) [file 13071_2016_1301_MOESM2_ESM.docx]

**Additional file 2:**

**Table S1. Nitric Oxide (NO) production of oxadiazole-2-oxides with SjTGR + NADPH or with SjTGR alone, or with worm homogenate supernatant**

| Compound | NO production (μM) | | |
| --- | --- | --- | --- |
|  | SjTGR | SjTGR + NADPH | Worm homogenate supernatant |
| Vehicle | 5.8±0.9 | 7.0±1.1 | 13.5±1.9 |
| PZQ | 7.2±0.4 | 7.3±0.8 | 12.1±2.1 |
| 4b | 3.8±0.7 | 6.5±0.9 | 7.6±0.7 |
| 7c | 6.0±0.1 | 9.2±0.1 | 10.9±1.2 |
| 13 | 5.2±0.3 | 7.5±1.0 | 8.6±1.1 |
| 16 | 2.5±0.4 | 5.0±0.3 | 9.4±1.2 |
| 20 | 4.0±0.5 | 6.3±0.5 | 7.8±1.1 |
| 21 | 4.8±0.4 | 5.0±0.3 | 9.2±1.8 |
| 22 | 4.2±0.3 | 7.3±1.5 | 7.5±0.5 |
| 23 | 3.2±0.1 | 3.5±0.3 | 7.7±1.4 |
| 26 | 3.0±0.8 | 6.2±1.2 | 9.8±1.8 |

NO production of compounds versus vehicle; analyzed by Student’s t-test, *P*>0.05.
