# Supplementary material for: Oxadiazole-2-oxides may have other functional targets, in addition to SjTGR, through which they cause mortality in Schistosoma japonicum
Source: Parasit Vectors. 2016 Jan 20;9:26. doi: 10.1186/s13071-016-1301-3 (PMC4721062; doi:10.1186/s13071-016-1301-3)
Supplement: Additional file 3: Table S2. — Killing activity on juvenile S. japonicum in vitro by oxadiazole-2-oxides. (DOCX 27 kb) [file 13071_2016_1301_MOESM3_ESM.docx]

**Additional file 3:**

**Table S2. Killing activity on juvenile *S. japonicum* in vitro by oxadiazole-2-oxides**

| Comp | Conc (μM)^a^ | Killing activity^b^ | | | |
| --- | --- | --- | --- | --- | --- |
|  |  | 24hr | 48hr | | 72hr |
| Vehicle | - | 9.0%D | 14.9%D | | 18.0%D |
| AS | 2 | 16.2%D | 24.3%D | | 32.4%D |
|  | 5 | 34.1%D | 36.4%D | | 40.9%D |
|  | 10 | 47.6%D | 66.7%D | | 71.4%D |
|  | 50 | 51.3%D | 66.7%D | | 76.9%D |
| 4a | 2 | 29.4%D | 35.3%D | | 44.1%D |
|  | 5 | 36.2%D | 48.9%D | | 53.2%D |
|  | 10 | 26.7%D | 37.8%D | | 58.9%D |
|  | 50 | 56.8%D | 88.6%D | | 90.9%D |
| 4b | 2 | 35.2%D | 35.2%D | | 42.3%D |
|  | 5 | 42.9%D | 46.9%D | | 51.0%D |
|  | 10 | 48.0%D | 50.0%D | | 60.0%D |
|  | 50 | 76.5%D | 88.2%D | | 100.0%D |
| 4c | 2 | 15.0%D | 20.0%D | | 20.0%D |
|  | 5 | 24.5%D | 24.5%D | | 26.4%D |
|  | 10 | 7.1%D | 14.3%D | | 17.9%D |
|  | 50 | 25.8%D | 25.8%D | | 51.6%D |
| 7a | 2 | 14.6%D | 29.2%D | | 41.7%D |
|  | 5 | 15.7%D | 39.2%D | | 39.2%D |
|  | 10 | 36.7%D | 36.7%D | 60.0%D | |
|  | 50 | 46.2%D | 53.3%D | | 70.0%D |
| 7b | 2 | 16.0%D | 26.6%D | | 31.9%D |
|  | 5 | 21.1%D | 26.3%D | | 36.8%D |
|  | 10 | 23.8%D | 33.3%D | | 57.1%D |
|  | 50 | 47.5%D | 55.9%D | | 57.6%D |
| 7c | 2 | 100.0%D | 100.0%D | | 100.0%D |
|  | 5 | 100.0%D | 100.0%D | | 100.0%D |
|  | 10 | 100.0%D | 100.0%D | | 100.0%D |
|  | 50 | 100.0%D | 100.0%D | | 100.0%D |
| 8 | 2 | 12.0%D | 18.0 %D | | 21.0%D |
|  | 5 | 12.8%D | 23.0%D | | 46.2%D |
|  | 10 | 15.1%D | 23.3%D | | 25.6%D |
|  | 50 | 26.8%D | 28.2%D | | 33.8%D |
| 9 | 2 | 17.6%D | 32.3%D | | 38.2%D |
|  | 5 | 31.3%D | 31.3%D | | 41.7%D |
|  | 10 | 39.0%D | 57.1%D | | 57.1%D |
|  | 50 | 100.0%D | 100.0%D | | 100.0%D |
| 10 | 2 | 21.3%D | 25.5%D | | 36.2%D |
|  | 5 | 37.0%D | 45.7%D | | 52.2%D |
|  | 10 | 30.3%D | 37.9%D | | 42.4%D |
|  | 50 | 69.0%D | 85.0%D | | 90.8%D |
| 11 | 2 | 26.3%D | 29.3%D | | 35.4%D |
|  | 5 | 38.3%D | 43.3%D | | 50.0%D |
|  | 10 | 31.6%D | 50.0%D | | 50.0%D |
|  | 50 | 60.6%D | 63.8%D | | 84.0%D |
| 12 | 2 | 17.3%D | 17.3%D | | 19.2%D |
|  | 5 | 16.4%D | 18.2%D | | 23.6%D |
|  | 10 | 21.3%D | 21.3%D | | 28.8%D |
|  | 50 | 33.3%D | 36.2%D | | 72.5%D |
| 13 | 2 | 13.0%D | 13.0%D | | 13.0%D |
|  | 5 | 14.2%D | 15.5%D | | 15.5%D |
|  | 10 | 19.0%D | 19.0%D | | 20.3%D |
|  | 50 | 23.3%D | 29.9%D | | 41.0%D |
| 14 | 2 | 21.6%D | 31.1%D | | 35.1%D |
|  | 5 | 26.4%D | 33.3%D | | 38.9%D |
|  | 10 | 30.4%D | 69.6%D | | 69.6%D |
|  | 50 | 46.4%D | 66.4%D | | 80.0%D |
| 15 | 2 | 21.3%D | 26.7%D | | 26.7%D |
|  | 5 | 35.1%D | 35.1%D | | 35.1%D |
|  | 10 | 32.0%D | 45.3%D | | 48.0%D |
|  | 50 | 57.1%D | 57.1%D | | 100.0%D |
| 16 | 2 | 14.7%D | 22.7%D | | 25.3%D |
|  | 5 | 16.1%D | 21.9%D | | 34.3%D |
|  | 10 | 20.9%D | 27.3%D | | 39.1%D |
|  | 50 | 31.4%D | 60.5%D | | 72.1%D |
| 17 | 2 | 28.1%D | 37.5%D | | 46.9%D |
|  | 5 | 29.2%D | 41.7%D | | 50.0%D |
|  | 10 | 40.0%D | 48.6%D | | 54.3%D |
|  | 50 | 45.8%D | 60.4%D | | 72.9%D |
| 18 | 2 | 24.1%D | 29.9%D | | 35.6%D |
|  | 5 | 33.8%D | 40.0%D | | 47.5%D |
|  | 10 | 36.6%D | 42.7%D | | 42.7%D |
|  | 50 | 52.4%D | 88.1%D | | 100.0%D |
| 19 | 2 | 15.1%D | 15.1%D | | 17.0%D |
|  | 5 | 12.5%D | 19.2%D | | 28.8%D |
|  | 10 | 43.8%D | 50.0%D | | 65.0%D |
|  | 50 | 82.5%D | 92.8%D | | 100.0%D |
| 20 | 2 | 8.5%D | 14.2%D | | 19.9%D |
|  | 5 | 20.0%D | 24.0%D | | 34.0%D |
|  | 10 | 43.4%D | 45.3%D | | 52.8%D |
|  | 50 | 45.6%D | 48.5%D | | 67.6%D |
| 21 | 2 | 22.5%D | 22.5%D | | 27.5%D |
|  | 5 | 26.7%D | 26.7%D | | 30.0%D |
|  | 10 | 30.0%D | 33.3%D | | 56.7%D |
|  | 50 | 33.3%D | 38.3%D | | 67.0%D |
| 22 | 2 | 18.1%D | 30.1%D | | 41.0%D |
|  | 5 | 18.6%D | 30.2%D | | 41.8%D |
|  | 10 | 134.5%D | 41.4%D | | 69.0%D |
|  | 50 | 100.0%D | 100.0%D | | 100.0%D |
| 23 | 2 | 6.0%D | 16.1%D | | 17.7%D |
|  | 5 | 9.0%D | 15.0%D | | 20.0%D |
|  | 10 | 45.0%D | 60.0%D | | 66.7%D |
|  | 50 | 69.9%D | 70.8%D | | 77.0%D |
| 24 | 2 | 27.0%D | 35.1%D | | 35.1%D |
|  | 5 | 27.9%D | 37.2%D | | 38.5%D |
|  | 10 | 40.4%D | 44.7%D | | 48.9%D |
|  | 50 | 66.7%D | 83.3%D | | 92.6%D |
| 25 | 2 | 8.3%D | 10.0%D | | 11.7%D |
|  | 5 | 16.0%D | 18.0%D | | 26.0%D |
|  | 10 | 27.4%D | 35.7%D | | 36.9%D |
|  | 50 | 42.9%D | 52.9%D | | 57.1%D |
| 26 | 2 | 36.4%D | 36.4%D | | 50.9%D |
|  | 5 | 48.9%D | 48.9%D | | 53.2%D |
|  | 10 | 56.1%D | 61.0%D | | 80.5%D |
|  | 50 | 46.3%D | 63.4%D | | 85.4%D |
| 27 | 2 | 9.1%D | 9.1%D | | 10.6%D |
|  | 5 | 14.9%D | 25.7%D | | 27.0%D |
|  | 10 | 15.6%D | 24.4%D | | 30.0%D |
|  | 50 | 40.0%D | 44.4%D | | 46.7%D |
| 28 | 2 | 8.2%D | 13.7%D | | 19.2%D |
|  | 5 | 14.8%D | 21.6%D | | 26.1%D |
|  | 10 | 14.5%D | 17.4%D | | 30.4%D |
|  | 50 | 31.9%D | 36.2%D | | 39.1%D |
| 29 | 2 | 30.0%D | 30.0%D | | 30.0%D |
|  | 5 | 30.0%D | 32.5%D | | 40.0%D |
|  | 10 | 30.2%D | 31.7%D | | 65.1%D |
|  | 50 | 42.3%D | 51.9%D | | 75.0%D |

^a^ The concentration of the chemicals on juvenile *S. japonicum* in vitro

^b^ Data collected by visual examination of worm movement and shape; % D = The number of worms dead / The total number of worms observed, and worms dead judged by unclear internal structure of juvenile worms with uncompleted tegument, and contents overflowing radially, or unclear internal structure with complete tegument, but having no motor activity during 1 min of continuous observation. The data presented are the average of three independent experiments.
