# Supplementary material for: Oxadiazole-2-oxides may have other functional targets, in addition to SjTGR, through which they cause mortality in Schistosoma japonicum
Source: Parasit Vectors. 2016 Jan 20;9:26. doi: 10.1186/s13071-016-1301-3 (PMC4721062; doi:10.1186/s13071-016-1301-3)
Supplement: Additional file 4: Table S3. — Killing activity on adult S. japonicum in vitro by oxadiazole-2-oxides. (DOCX 23 kb) [file 13071_2016_1301_MOESM4_ESM.docx]

**Additional file 4:**

**Table S3. Killing activity on adult *S. japonicum* *in vitro* by oxadiazole-2-oxides**

| Comp | Killing activity^b^ | | | |
| --- | --- | --- | --- | --- |
|  | Conc (μM)^a^ | 24hr | 48hr | 72hr |
| Vehicle |  | n.e. | n.e. | n.e. |
| PZQ | 10 | 25.0%D | 25.0%D | 37.5%D |
|  | 25 | 25.0%D | 25.0%D | 62.5%D |
|  | 50 | 25.0%D | 37.5%D | 62.5%D |
|  | 100 | 37.5%D | 50.0%D | 87.5%D |
| 4a | 10 | 37.5%D | 37.5%D | 50.0%D |
|  | 25 | 50.0%D | 100.0%D | 100.0%D |
|  | 50 | 88.9%D | 100.0%D | 100.0%D |
|  | 100 | 100.0%D | 100.0%D | 100.0%D |
| 4b | 10 | n.e. | n.e. | n.e. |
|  | 25 | 63.6%D | 81.8%D | 66.7%D |
|  | 50 | 60.0%D | 80.0%D | 100.0%D |
|  | 100 | 57.1%D | 100.0%D | 100.0%D |
| 4c | 10 | n.e. | n.e. | n.e. |
|  | 25 | n.e. | n.e. | n.e. |
|  | 50 | n.e. | n.e. | n.e. |
|  | 100 | 57.1%D | 71.4%D | 71.4%D |
| 7a | 10 | 70.0%D | 100.0%D | 100.0%D |
|  | 25 | 88.9%D | 100.0%D | 100.0%D |
|  | 50 | 87.5%D | 100.0%D | 100.0%D |
|  | 100 | 100.0%D | 100.0%D | 100.0%D |
| 7b | 10 | 16.7%D | 50.0%D | 83.3%D |
|  | 25 | 100%D | 100%D | 100%D |
|  | 50 | 100%D | 100%D | 100%D |
|  | 100 | 100%D | 100%D | 100%D |
| 7c | 10 | n.e. | 70.0%D | 70.0%D |
|  | 25 | 77.8%D | 100.0%D | 100.0%D |
|  | 50 | 100.0%D | 100.0%D | 100.0%D |
|  | 100 | 100.0%D | 100.0%D | 100.0%D |
| 8 | 10 | n.e. | 57.1%D | 71.4%D |
|  | 25 | 50.0%D | 66.7%D | 100%D |
|  | 50 | 77.8%D | 88.9%D | 100%D |
|  | 100 | 100%D | 100%D | 100%D |
| 9 | 10 | sluggish | 50.0%D | 83.3%D |
|  | 25 | 60.0%D | 100%D | 100%D |
|  | 50 | 66.7%D | 100%D | 100%D |
|  | 100 | 100%D | 100%D | 100%D |
| 10 | 10 | sluggish | sluggish | 42.9%D |
|  | 25 | sluggish | 100%D | 100%D |
|  | 50 | 50.0%D | 100%D | 100%D |
|  | 100 | 75.0%D | 100%D | 100%D |
| 11 | 10 | n.e. | sluggish | 62.5%D |
|  | 25 | n.e. | 50.0%D | 80.0%D |
|  | 50 | 66.7%D | 66.7%D | 100%D |
|  | 100 | 100%D | 100%D | 100%D |
| 12 | 10 | sluggish | sluggish | 55.6%D |
|  | 25 | 80.0%D | 100%D | 100%D |
|  | 50 | 88.9%D | 100%D | 100%D |
|  | 100 | 100%D | 100%D | 100%D |
| 13 | 10 | sluggish | 90.0%D | 100%D |
|  | 25 | sluggish | 100%D | 100%D |
|  | 50 | 100%D | 100%D | 100%D |
|  | 100 | 100%D | 100%D | 100%D |
| 14 | 10 | 33.3%D | 44.4%D | 77.8%D |
|  | 25 | 75.0%D | 87.5%D | 100%D |
|  | 50 | 100%D | 100%D | 100%D |
|  | 100 | 100%D | 100%D | 100%D |
| 15 | 10 | sluggish | 88.9%D | 100%D |
|  | 25 | 50.0%D | 100%D | 100%D |
|  | 50 | 70.0%D | 100%D | 100%D |
|  | 100 | 87.5%D | 100%D | 100%D |
| 16 | 10 | n.e. | 50.0%D | 80.0%D |
|  | 25 | 50.0%D | 75.0%D | 100.0%D |
|  | 50 | 100.0%D | 100.0%D | 100.0%D |
|  | 100 | 100.0%D | 100.0%D | 100.0%D |
| 17 | 10 | n.e. | n.e. | 50.0%D |
|  | 25 | n.e. | n.e. | 62.5%D |
|  | 50 | n.e. | 40.0%D | 87.5%D |
|  | 100 | sluggish | 57.1%D | 85.7%D |
| 18 | 10 | 16.7%D | 16.7%D | 50.0%D |
|  | 25 | 75.0%D | 87.5%D | 100%D |
|  | 50 | 100%D | 100%D | 100%D |
|  | 100 | 100%D | 100%D | 100%D |
| 19 | 10 | 80.0%D | 100%D | 100%D |
|  | 25 | 100%D | 100%D | 100%D |
|  | 50 | 100%D | 100%D | 100%D |
|  | 100 | 100%D | 100%D | 100%D |
| 20 | 10 | n.e. | 50.0%D | 75.0%D |
|  | 25 | 50.0% | 100%D | 100%D |
|  | 50 | 70.0%D | 100%D | 100%D |
|  | 100 | 100%D | 100%D | 100%D |
| 21 | 10 | n.e. | sluggish | sluggish |
|  | 25 | n.e | 100%D | 100%D |
|  | 50 | 66.7%D | 100%D | 100%D |
|  | 100 | 75.0%D | 100%D | 100%D |
| 22 | 10 | 44.4%D | 66.7%D | 88.9%D |
|  | 25 | 90.0%D | 100.0%D | 100.0%D |
|  | 50 | 90.9%D | 100.0%D | 100.0%D |
|  | 100 | 100.0%D | 100.0%D | 100.0%D |
| 23 | 10 | 62.5%D | 87.5%D | 100%D |
|  | 25 | 100%D | 100%D | 100%D |
|  | 50 | 100%D | 100%D | 100%D |
|  | 100 | 100%D | 100%D | 100%D |
| 24 | 10 | 62.5%D | 75.0%D | 100%D |
|  | 25 | 100%D | 100%D | 100%D |
|  | 50 | 100%D | 100%D | 100%D |
|  | 100 | 100%D | 100%D | 100%D |
| 25 | 10 | n.e. | n.e. | n.e |
|  | 25 | n.e. | n.e. | sluggish |
|  | 50 | n.e. | sluggish | 40.0%D |
|  | 100 | 42.9%D | 57.1%D | 85.7%D |
| 26 | 10 | n.e. | n.e. | 16.7%D |
|  | 25 | 85.7%D | 100%D | 100%D |
|  | 50 | 100%D | 100%D | 100%D |
|  | 100 | 100%D | 100%D | 100%D |
| 27 | 10 | n.e. | n.e. | 62.5%D |
|  | 25 | sluggish | sluggish | 75.0%D |
|  | 50 | sluggish | sluggish | 87.5%D |
|  | 100 | 22.2%D | 33.3%D | 77.8%D |
| 28 | 10 | 57.1%D | 57.1%D | 71.4%D |
|  | 25 | 100%D | 100%D | 100%D |
|  | 50 | 100%D | 100%D | 100%D |
|  | 100 | 100%D | 100%D | 100%D |
| 29 | 10 | n.e. | n.e. | n.e. |
|  | 25 | n.e. | n.e. | sluggish |
|  | 50 | n.e. | sluggish | 16.7%D |
|  | 100 | n.e. | sluggish | 66.7%D |

^a^ The concentration of the chemicals on adult *S. japonicum* in vitro

^b^ Data collected by visual examination of worm movement and shape; n.e. (no effect): all worms are scored as active in culture with typical appearance; sluggish: worm movement is significantly reduced; % D = The number of worms dead / The total number of worms observed, and worms dead judged by lack of movement in 2 minutes as well as morphological and tegumental alterations. The data presented are the average of three independent experiments.
